# Basal Temperature Measurement Using a Multi-Sensor Armband in Australian Young Women: A Comparative Observational Study

**DOI:** 10.2196/mhealth.4263

**Published:** 2015-10-05

**Authors:** John D Wark, Lucy Henningham, Alexandra Gorelik, Yasmin Jayasinghe, Stefanie Hartley, Suzanne Marie Garland

**Affiliations:** ^1^University of MelbourneDepartment of MedicineRoyal Melbourne HospitalParkvilleAustralia; ^2^Royal Melbourne HospitalBone and Mineral MedicineParkvilleAustralia; ^3^University of MelbourneMelbourne EpiCentreRoyal Melbourne HospitalParkvilleAustralia; ^4^University of MelbourneDepartment of Obstetrics and GynaecologyParkvilleAustralia; ^5^Murdoch Childrens Research InstituteInfection and Immunity ThemeParkvilleAustralia; ^6^Royal Women's HospitalDepartment of Microbiology & Infectious DiseasesParkvilleAustralia

**Keywords:** basal body temperature, young female health initiative, BodyMedia SenseWear, ovulation, menstrual cycle, young women

## Abstract

**Background:**

The menstrual cycle is a key marker of health in women of reproductive age. Monitoring ovulation is useful in health studies involving young women. The upward shift in basal body temperature, which occurs shortly after ovulation and continues until the next menses, is a potentially useful marker of ovulation, which has been exploited in clinical and research settings.

**Objective:**

We investigated the utility of BodyMedia SenseWear (BMSW) in monitoring ovulation in young women by analyzing the correlation and agreement of basal temperatures measured using BMSW and a digital oral thermometer.

**Methods:**

Kappa statistics were used to determine the agreement in ovulation detection between the two devices, for each participant, under each form of analysis. Participants also completed an online questionnaire assessing the acceptability of both devices.

**Results:**

We recruited 16 participants with 15 of them providing analyzable data (11 OCP non-users, 4 OCP users). Weak to moderate correlations were observed between thermometer and BMSW temperature measurements averaged over 5 different time intervals. However, no agreement between methods was observed using Bland-Altman plots. There was a significant difference in the range of temperatures that each device recorded (thermometer: 35.3-37.2°C, BMSW: 29.7-36.7°C) with BMSW temperatures significantly lower than thermometer temperatures: mean 34.6°C (SD 1.2) versus 36.4°C (SD 0.3) respectively, *P*<.001. Poor agreement was observed between devices under quantitative analysis of ovulation while fair agreement was observed under visual analysis. Under both quantitative and visual analysis, there was 0% agreement for evidence of ovulation.

**Conclusions:**

This study demonstrated the importance of evaluating biomeasures collected using mobile monitoring devices by comparison with standard methods. It revealed a relatively poor correlation between BMSW and oral thermometer temperature readings and suggested that BMSW is unlikely to detect an upward shift in basal body temperature. Participant behavior suggested poor compliance in the use of BMSW for basal temperature measurement and that the basal body temperature method may not be suitable for use in unselected samples of young women. There is a need for research tools for monitoring ovulation that are simple, self-administered, and inexpensive, yet appealing to young women.

## Introduction

The menstrual cycle is one of the key and characteristic physiological processes of women and is an important indicator of overall health in women of reproductive age [1]. Continuous fluctuations in hormone levels result in observable physiological changes throughout the menstrual cycle. These include alterations in urine luteinizing hormone (LH levels) [2,3], cervical mucus [2,4] and basal body temperature (BBT) [2,5,6]. The cyclic nature of the menstrual cycle allows for the observed presence or absence of these physiological alterations to be used as indicators of ovarian function [2]. BBT is defined as “the waking temperature of the body before any activity” [5]. Generally in women with ovulatory cycles, an increase in BBT within the range of 0.2-0.5°C occurs shortly after ovulation and persists until the following menses [5,7-9]. Thus, BBT is considered biphasic with the temperature shift generally regarded as confirmation of ovulation [5,7,8]. The relationship between the menstrual cycle and fluctuations in body temperature was first observed in 1867 [6,8]. It was not until 1926, however, that a direct association between this temperature shift and ovulation was determined [6]. Since then, this biphasic shift in BBT has been used clinically, in research, and by individuals, in various contexts including achieving pregnancy, contraception, investigation of infertility, and as a general indicator of ovarian function.

Despite modern technological advancements, the most frequently used method for monitoring BBT in both research and self-assessment settings is via a thermometer, as it has been for decades [5]. Originally this involved oral, rectal, or vaginal application of a mercury thermometer. However, due to health concerns associated with mercury and the invention of digital thermometers, the currently recommended procedure is for women to take their temperature immediately after waking using an oral digital thermometer [5].

Although the BBT method has significant limitations, it is simple, non-invasive, and cheap. It therefore continues to be useful for some clinical and research applications. Self-plotted and visually assessed temperature has been reported to be inaccurate [10]. However, interpretation using the quantitative mean temperature method (MTM) of Vollman appears to appreciably improve the method’s reliability in detecting ovulation [8]. Nevertheless, it is also important that any such method achieves a high level of acceptability and compliance among users, and it would also be advantageous if other physiological data could be conveniently collected concurrently for various clinical and research purposes.

**Figure 1:**
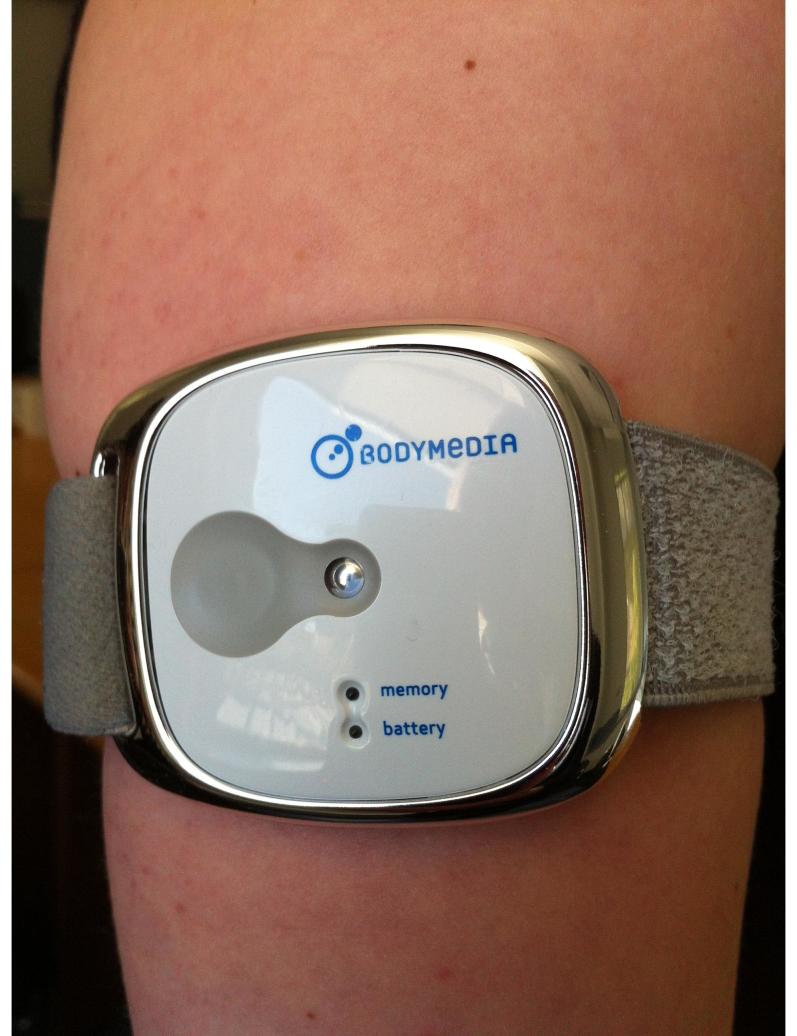
The BodyMedia SenseWear armband as worn.

The BodyMedia Inc. armbands combine four sensors, all of which can monitor a variety of physiological parameters over time. BodyMedia SenseWear (BMSW; Figure 1) is a research model, and BodyMedia FIT is a consumer model. The sensors include a thermistor-based sensor to measure skin temperature. It has been proposed that continuously measured skin temperature is linearly reflective of core body temperature [11]. Thus, we hypothesized that, when worn under basal conditions, BMSW would be indicative of BBT and BMSW would be able to detect the upward shift in BBT that occurs shortly after ovulation. Should that be so, the BMSW and similar devices could be an accurate and reliable alternative to the current standard BBT monitoring device (a digital oral thermometer), also enabling a range of physiological data to be collected simultaneously.

Our first aim was to compare concurrent basal temperature measurements taken using an oral digital thermometer (the criterion method) with skin temperature recorded using the BMSW. For this purpose, we chose to study a sample of healthy young women aged 18-25 years who had been recruited for a wide-ranging study of young women’s health, the Young Female Health Initiative (YFHI) [12].

## Methods

The BBT study was designed as a substudy of the YFHI Launch Study. The YFHI Launch study is a multidisciplinary investigation of young women’s health, utilizing modern information and communication technology, a self-administered online questionnaire, and a health check carried out at the study site.

### Preliminary Study

A preliminary study was conducted to determine an effective and acceptable methodology for collecting basal skin temperatures using BMSW. Two methodologies were proposed. The first involved participants wearing BMSW for 20 minutes each morning immediately after waking and before undertaking any form of activity. At the commencement of the 20-minute interval, participants were also asked to take their temperature using a digital oral thermometer. The second methodology involved participants wearing BMSW overnight and removing it immediately on waking. Participants were also requested to complete an online feedback questionnaire reflecting on acceptability of the different methodologies. This preliminary study was conducted on a convenience sample of 9 female volunteers aged 20-24 years, who were not included in the main BBT study. It was found that wearing BMSW for 20 minutes immediately after waking showed a continuous increase in temperature from start to end of the 20-minute period, for all participants on all days (data not shown). Hence, 20 minutes was deemed an insufficient period of time for BMSW to register a stable temperature reading. As anticipated, the BMSW recorded relatively stable skin temperatures on waking when the device was worn all night. Therefore, the overnight method was chosen for use in this study.

### Participants

Young women aged 18-25 years, living in the State of Victoria, Australia, and not using any hormonal form of contraception were eligible to participate in the BBT study. Exclusion criteria included (1) known diagnosis of disorders causing amenorrhea/anovulation and (2) current use of hormonal contraceptives. Age-matched subjects taking combined oral contraceptive pills (OCP) were also recruited and formed a control group. Participants were recruited from three sources: (1) expressions of interest submitted through the YFHI website, (2) participants who had completed the Vaccine Against Cervical Cancer Impact and Effectiveness Study (VACCINE) [13], a study measuring the Australian human papillomavirus (HPV) vaccine program effectiveness in vaccine eligible participants, and (3) current YFHI Launch Study participants who were recruited prior to the introduction of the BBT study. All participants sourced via their expression of interest through the YFHI website were notified via email by YFHI staff. Participants sourced through either VACCINE or the YFHI Launch Study were recruited using participant lists generated from the respective studies’ databases. All potential participants contacted from VACCINE and the YFHI Launch Study had given consent to be contacted in the future about other studies for which they may be eligible.

### Telephone Screening

Telephone screening was performed to provide participants with an overview of the study and to assess participant eligibility. Age, current address, and use of hormonal contraception were assessed and verbal informed consent obtained. Participants were also asked about their height, weight, handedness, and approximate nightly bedtime hours to set up BMSW.

### Study Procedures

Eligible participants were sent a study package containing a welcome letter thanking the participant for their participation and explaining the contents of the package, an instruction booklet, the BMSW armband and charging cable (Temple Healthcare), an Omron model MC-246 digital oral thermometer (Chemist Warehouse), reply-paid and registered postal labels, a paper log for those who chose this method of recording their temperatures taken using the thermometer, and a participant information and consent document to be completed by participants. Delivery was timed so that participants would receive their study package approximately 1 week before their next menstrual period was due, to ensure they were able to commence the temperature measuring on the first day of their period. Participants were requested to return the BMSW armband and charger and, for those applicable, the paper log at the completion of the study.

Participants were asked to commence measuring their temperature on the first day of their menstrual period and to do so every day until the first day of their following period. Participants were instructed to put the armband on immediately before going to bed and to sleep wearing it every night for the duration of the chosen menstrual cycle. They were asked to remove it immediately after waking up and before getting out of bed the following morning. Correct use of BMSW involved wearing the armband with the monitor placed on the back of the left upper arm, with the armband automatically turning itself on upon making contact with one’s skin.

Participants were given standard instructions for obtaining BBT: to use the thermometer orally every morning according to the manufacturer’s instructions immediately after waking and before performing any form of activity, including getting out of bed or consuming any food or drink. Participants were instructed to refrain from removing the BMSW armband until after they had used the thermometer, in order to ensure that the temperatures obtained from the two devices were comparable.

Participants who owned a smartphone were asked to download the WomanLog Pro app to record temperatures taken using the thermometer. This app is a menstrual cycle calendar, with a BBT recording and charting function. Participants were asked to submit their recorded temperatures by email at the conclusion of data collection. All temperatures recorded using the WomanLog Pro app were exported by a researcher (LH) directly from their cycle overview into Microsoft Excel in order to allow the researcher to graph data. Similarly, results recorded into paper logs were entered by the researcher into a Microsoft Excel spreadsheet before being plotted on graphs. Participants were given the option to use the app or a paper log. If participants noticed anything irregular with their menstrual cycle or forgot to wear BMSW one night or take their temperature one morning using the thermometer, they were asked to record this using either the paper log or WomanLog Pro. Participants were also asked to record whether they experienced any intercurrent illness or fever and any irregular wakening times. At the completion of their menstrual cycle, participants were asked to complete a brief, online, self-administered feedback questionnaire, generated using SurveyMonkey. This questionnaire asked participants a series of questions encouraging them to reflect on the use of BMSW, the thermometer, and their most recent menstrual cycle. Responses were elicited using a 5-point Likert scale with answers ranging from “completely false” to “completely true”.

### Statistical Analysis

Upon return of the study package, all data were exported from BMSW into Microsoft Excel using SenseWear Professional Software 7.0 (BodyMedia Inc.). Daily wakening time for each participant was identified by BMSW and the mean temperatures for 10-, 30-, 60-, 90-, and 120-minute intervals prior to this wakening time were calculated.

Correlation between BMSW and thermometer temperatures was analyzed using Spearman’s rank correlation test, while the level of agreement between the temperatures obtained via the two methods was further investigated using a Bland-Altman (BA) plot [14].

Temperature charts were analyzed for evidence of ovulation according to standard BBT criteria. This was achieved through charting and analyzing temperatures taken using the thermometer and BMSW, which was performed both visually as well as quantitatively using MTM [15]. Visual analysis was performed independently by 2 or 3 observers, blinded to group allocation, following the criteria outlined in Table 1 [16]. The level of agreement between two methods in detecting possible ovulation and interrater reliability were determined using Kappa statistics.

All analyses were performed using STATA version 12 (StataCorp), and *P*<.05 was considered statistically significant.

The study protocol was reviewed scientifically and approved by the Royal Women’s Hospital Human Research and Ethics Committees.

**Table 1:** Outline of criteria used for evidence of ovulation when visually analyzing temperature charts of temperature recorded using the digital oral thermometer and BMSW.

	Criteria for evidence of ovulation for visual analysis of BBT charts
1. Biphasic^a^ by >0.2°C	Indicated by a 3-day sustained shift compared with 6 previous temperatures around expected time of ovulation, calculated 2 weeks prior to following menses
2. Adequate thermal shift^a^	Sustained for at least 11 days; fast enough rise (<2 days); absence of deep falls in the luteal phase
3. Presence of a nadir	Fall in temperature immediately prior to sustained temperature rise

^a^The presence of biphasic and thermal shift are necessary to say there is evidence of ovulation, while presence of a nadir is supportive.

## Results

### Recruitment and Participation

We recruited 24 young women. Of these, 16 participants returned their study package. Their mean age was 22.1 years (SD 1.7). Twelve of these were not currently using hormonal contraception, while 4 were currently using an OCP.

Varying levels of completeness of the study protocol were observed in the 16 participants. Four participants completed the study in full, while 11 completed at least 17 days of temperature measurements. One participant returned their study package after only 4 days, and her data were excluded from analysis. Hence, interpretable data allowing comparison of the temperature measurement methods were available in 15 participants (12 non-OCP users, 3 OCP user controls). All available data from these participants were used to compare the BMSW and the digital thermometer methods of temperature measurement.

### Comparison of Temperatures Recorded Using BodyMedia SenseWear and the Thermometer

**Figure 2:**
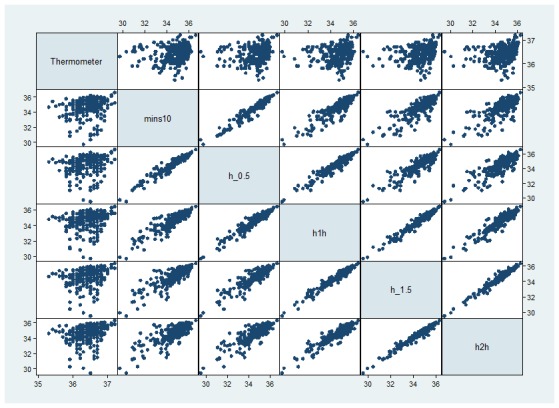
Correlations between thermometer and different time points performed to determine correlation between temperatures recorded by BMSW 10, 30, 60, 90, and 120 minutes before waking and the digital oral thermometer, as well as correlation between temperatures recorded at each of the 5 time intervals.

Weak-to-moderate correlations were observed between the thermometer and BMSW at the five time intervals (range of rho values .28-.4; Figure 2). However, strong intra-participant correlations were observed between the different time intervals (rho ranged from .76-.97, *P*<.001; Figure 2).

**Figure 3:**
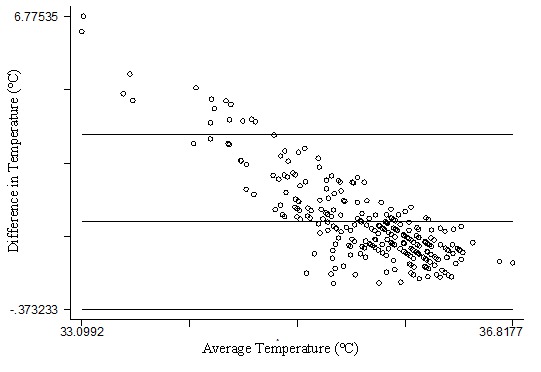
Bland-Altman comparison of thermometer and average temperature, for the 60-minute interval before waking recorded by BMSW (each dot represents one participant’s temperature readings using the thermometer and BMSW).

BA plots for all five time intervals showed no agreement with the thermometer, with a substantial level of variability and systematic bias (representative data for the 60-minute interval shown in Figure 3). The poor agreement between the thermometer and BMSW was similar for all five BMSW time intervals, indicated by the range of the mean difference between the thermometer at each of the time intervals (range 1.772-1.810). However, the negative slope of points on all these BA plots indicated a higher level of agreement between BMSW and the thermometer at higher temperatures. Thus, there was evidence of bias between recordings (temperature – BMSW) with a greater temperature differential being seen at lower mean temperatures.

A significant difference in the range of temperatures recorded by each device was also apparent. The range of temperatures measured using the thermometer was small (35.3-37.2°C), with all temperatures lying within the boundaries considered normal for core temperature [17]. The range of temperatures recorded using BMSW was much wider (29.7-36.7°C). Furthermore, the absolute values of temperatures recorded using BMSW were significantly lower than those recorded using the thermometer: mean 34.6°C (SD 1.2) versus 36.4°C (SD 0.3) respectively, *P*<.001.

### Evidence of Ovulation

The results of the analysis for evidence of ovulation for all four methods performed (visual thermometer, visual BMSW, quantitative thermometer, and quantitative BMSW) are outlined in Table 2. Table 3 contains the Kappa analysis, indicating the agreement between methods overall, as well as for detection of ovulation, anovulation, and inconclusive cases.

**Table 2:** Number of ovulatory, anovulatory, and inconclusive menstrual cycles detected by BMSW and the thermometer under visual^a^ and quantitative^b^ analysis.

Method	Ovulatory	Anovulatory	Inconclusive
Visual thermometer	1	11	3
Visual BMSW	1	8	5
Quantitative (MTM) thermometer	5	9	1
Quantitative (MTM) BMSW	0	14	1

^a^Mathematical analysis was performed using the quantitative MTM method on Microsoft Excel.

^b^Visual analysis was performed by LH and YJ using the criteria for ovulation in Table 1. When these observers disagreed, JDW also made a visual determination and the majority decision was accepted. Temperature charts for visual analysis were constructed using Microsoft Excel.

**Table 3:** Kappa analysis of agreements between BMSW and the thermometer under quantitative^a^ and visual analysis.

Methods	Kappa^b^	Agreement, %	Agreement for ovulation, %	Agreement for anovulation, %	Agreement for inconclusive cases, %
Quantitative BMSW vs quantitative thermometer	.0816	60.00	0.00	60.00	0.00
Visual BMSW vs visual thermometer	.4915	73.33	0.00	53.33	20.00
Quantitative BMSW vs visual BMSW	.1589	60.00	0.00	53.33	6.67
Quantitative thermometer vs visual thermometer	.3023	73.33	6.67	53.33	0.00

^a^Mathematical analysis was performed using the quantitative MTM method on Microsoft Excel.

^b^Kappa >.75 indicates excellent agreement, .4≤ kappa ≤.75 indicates fair to good agreement, and <.4 indicates moderate to poor agreement.

#### Visual Analysis of Ovulation

Visual analysis of temperatures taken using the thermometer deemed 1 participant ovulatory, 11 anovulatory, and 3 inconclusive (Table 2). Visual analysis of BMSW found 1 to be ovulatory, 8 anovulatory, and 5 inconclusive. The kappa statistic for these two methods was indicative of fair agreement, with agreement of 73%, which was the highest level of agreement observed between two methods assessing for evidence of ovulation. However, the single participant found to be ovulatory for each of these two methods was not the same, and thus the agreement for determination of ovulation was 0%.

#### Quantitative Analysis of Ovulation

Quantitative MTM analysis of temperatures taken using the thermometer found 5 participants to be ovulatory , 9 anovulatory, and 1 inconclusive, while quantitative MTM analysis of temperatures recorded using BMSW found 0 ovulatory, 14 anovulatory, and 1 inconclusive. The kappa statistic indicated poor agreement, with an agreement of 60% for these two methods. This was the lowest level of agreement observed. There was 0% agreement for determination of ovulation.

Twelve of 16 participants completed the online post-study feedback questionnaire (Table 4). Responses are described qualitatively below because of the small sample size. Responses clearly indicated that neither discomfort nor self-consciousness were concerns associated with BMSW use. Responses to questions in regards to convenience, however, were relatively inconclusive. When asked whether they found the device a convenient method for monitoring the menstrual cycle, the majority of participants gave neutral responses. Responses regarding the appeal of BMSW as a tool for monitoring ovulation were similarly inconclusive. However, there was a slight preference for the thermometer over BMSW. Interest in monitoring the menstrual cycle daily with devices such as BMSW or a thermometer was equivocal. However, participants expressed a strong interest in learning more about their menstrual cycle from BBT temperature monitoring.

**Table 4:** Summary of participant responses^a^ to questions asked on their experience using both the BMSW and the thermometer in the post data collection feedback questionnaire.

Statement	Scale of agreement: 1=“completely false” to 5=“completely true”					Median
	1	2	3	4	5	
1. “I often found the activity monitor painful to wear”	41.7% (5)	50.0%^b^ (6)	8.3% (1)	0.0% (0)	0.0% (0)	2
2. “I often found the activity monitor uncomfortable to wear”	8.3% (1)	50.0%^b^ (6)	25.0% (3)	16.7% (2)	0.0% (0)	2
3. “I often found the oral thermometer uncomfortable to use”	75.0%^b^ (9)	0.0% (0)	8.3% (1)	8.3% (1)	8.3% (1)	1
4. “I often found the oral thermometer painful to use”	91.7%^b^ (11)	8.3% (1)	0.0% (0)	0.0% (0)	0.0% (0)	1
5. “I often did not wear the activity monitor because it was uncomfortable or painful”	75.0%^b^ (9)	16.7% (2)	8.3% (1)	0.0% (0)	0.0% (0)	1
6. “I often did not use the thermometer because it was uncomfortable or painful”	100%^b^ (12)	0.0% (0)	0.0% (0)	0.0% (0)	0.0% (0)	1
7. “I found wearing the activity monitor overnight a hassle and interfered with my sleep”	33.3% (4)	58.3%^b^ (7)	8.3% (1)	0.0% (0)	0.0% (0)	2
8. “I found using the oral thermometer a hassle and interfered with my day”	75.0%^b^ (9)	8.3% (1)	8.3% (1)	8.3% (1)	0.0% (0)	1
9. “I often forgot to put the activity monitor on before going to sleep”	50.0%^b^ (6)	25.0% (3)	8.3% (1)	16.7% (2)	0.0% (0)	1.5
10. “I often forgot to use the oral thermometer first thing upon waking every morning”	58.3%^b^ (7)	33.3% (4)	8.3% (1)	0.0% (0)	0.0% (0)	1
11. “I found the activity monitor a convenient way to measure basal body temperature”	8.3% (1)	0.0% (0)	50.0%^b^ (6)	8.3% (1)	33.3% (4)	3
12. “I found the oral thermometer a convenient way to measure basal body temperature”	0.0% (0)	8.3% (1)	33.3%^b^ (4)	25% (3)	33.3%^b^ (4)	4
13. “I often felt self-conscious or embarrassed wearing the activity monitor every night to measure basal body temperature”	83.3%^b^ (10)	8.3% (1)	8.3% (1)	0.0% (0)	0.0% (0)	1
14. “I often felt self-conscious or embarrassed using the oral thermometer every morning to measure basal body temperature”	91.7%^b^ (11)	8.3% (1)	0.0% (0)	0.0% (0)	0.0% (0)	1
15. “I found the oral thermometer more convenient than the activity monitor to measure basal body temperature”	33.3%^b^ (4)	16.7% (2)	0.0% (0)	33.3%^b^ (4)	16.7% (2)	3
16. “I preferred using the activity monitor over the oral thermometer”	16.7% (2)	33.3%^b^ (4)	8.3% (1)	25% (3)	16.7% (2)	2.5
17. “I would like to use a device such as an oral thermometer or activity monitor every day in order to observe and keep track of my menstrual cycle”	33.3% (4)	8.3% (1)	41.7%^b^ (5)	8.3% (1)	8.3% (1)	3
18. “I would prefer to record my menstrual cycle observations by completing a survey rather than wearing an activity monitor every night”	0.0% (0)	33.3% (4)	58.3%^b^ (7)	8.3% (1)	0.0% (0)	3
19. “I would prefer to record my menstrual cycle observations by completing a survey rather than using an oral thermometer every morning”	0.0% (0)	33.3%^b^ (4)	33.3%^b^ (4)	33.3%^b^ (4)	0.0% (0)	3
20. “I am interested in learning more about my menstrual cycle based on the basal body temperature tracking I have just completed”	16.7% (2)	0.0% (0)	8.3% (1)	16.7% (2)	58.3%^b^ (7)	5

^a^Responses were elicited using a 5-point Likert Scale. The number of responses in each category is given in parentheses. The median response score for all questions is also included.

^b^The most common response scores for each statement.

## Discussion

### Principal Findings

This study illustrated the importance of evaluating biomeasures derived from mobile monitoring devices by comparison with standard measuring methods. In particular, there were only modest correlations and poor agreement between basal temperatures measured using the criterion method (digital oral thermometer) and the BMSW device. Results from the Spearman’s correlation test and BA plots refuted the main hypothesis, demonstrating very modest correlation and agreement between temperatures taken using the thermometer and BMSW over any time interval. Although generally weak correlations were found between the thermometer and BMSW, there was a strong correlation of BMSW temperature averages between each of the five time intervals. This demonstrates consistent temperature measurement by BMSW’s skin temperature sensor, which suggests the potential for high precision and reproducibility from the device. As data from the preliminary study indicated, however, this performance can be achieved only after BMSW has had sufficient time to stabilize, which is a wearing interval of greater than 20 minutes. We chose to test a wearing period of 20 minutes because we reasoned that participants were unlikely to comply with longer wearing periods first thing each morning before rising, for a whole menstrual cycle.

Despite the apparent potential for high temperature-measuring precision and consistent data, the wide range of temperatures measured by BMSW both within and between participants suggests, as with any external measurement, that it can be affected by environmental and perhaps individual physiological factors. Study participation occurred from June-September, correlating with the Australian winter. It is possible that different levels and mechanisms of heating were used by different participants or on different occasions. The wide range is also likely to have been caused by confounding factors such as not wearing the device correctly on some nights, variation in bedding and sleepwear used, alcohol consumption, variability in the time when the temperature was taken, or variability in sleep duration. Participants were asked to record whether they fell ill throughout the course of the study or had abnormal waking times, which are two potential effectors of skin temperature. However, information was not available relating to other possible confounding factors, such as whether they slept in the same room or whether they slept alone or with a partner during the study. This demonstrates the difficulty in controlling studies involving body temperature, which is an issue not only presenting itself now with BMSW or skin temperature but is repeatedly observed when using the BBT method [5].

This study aimed to evaluate the potential of mobile monitoring devices such as BMSW to obtain evidence of ovulation by detecting the upward shift in BBT that accompanies this important physiological event. Given suggestions that continuously measured skin temperature, a function BMSW can perform, is linearly reflective of core body temperature [11], it was hypothesized that, under basal conditions, BMSW would be comparable to the traditional method of a thermometer in detecting the upward shift in BBT.

However, given the modest correlation that we found between the benchmark clinical method of digital oral basal temperature measurement and BMSW-determined basal skin temperature measurement, it seems unlikely that the BMSW is a suitable device for clinical monitoring of ovulation in a population of young women such as we studied. We cannot draw more definitive conclusions on this point for several reasons. First, only a minority of participants (25%) completed data collection for a full menstrual cycle. Second, standard quantitative clinical criteria for ovulation (quantitative mean temperature method [15]) were met in only one third of participants, which is appreciably lower than would have been predicted in such a population [18]. Quantitative and visual analysis of charts of the temperatures recorded by the thermometer and BMSW over the course of each participant’s menstrual cycle provided little support for the main hypothesis. When analyzed quantitatively, BMSW showed very poor agreement with the thermometer (kappa=.0816, agreement=60%). A notable observation from the quantitative analysis was BMSW’s inability to detect evidence for ovulation in any participant, while thermometer temperatures deemed 5 participants ovulatory.

Patterns of participant behavior suggest low compliance with the BBT method. For instance, a high number of participants frequently omitted taking their temperature throughout the study or did not commence the study. Thus, it is possible that the apparently small number of ovulatory participants is the result of low participant compliance and the subsequent incorrect and ineffective use of the BBT method. Hence, our findings suggest that the methods evaluated may not be suitable for the monitoring of menstrual cycles or documenting ovulation in the demographic studied. It may be that outcomes would be better in a sample of young women more motivated to document their menstrual cycles, for example, due to a desire to achieve pregnancy.

### Strengths and Limitations

Strengths of this study include its novelty: limited research has been carried out on the use of continuously measured skin temperature in monitoring ovulation. Moreover, although validated in many other areas of health, at the time of this study no research had been published on the use of BMSW in detecting ovulation or investigating reproductive health in general. The data comparing oral digital thermometer measurement of basal temperature with the use of BMSW temperature readings provided strong if not conclusive evidence that BMSW data were unlikely to be useful for basal temperature monitoring across the menstrual cycle. Limitations of the study included the volunteer nature of the sample studied, so that findings could not be generalized to the entire population of young Australian women, the inability to monitor independently that participants followed all aspects of the study protocol, and that the number of participants completing data collection did not allow an adequate evaluation of the ability of the methods tested to detect ovulation. Another limitation of the study was the lack of more sensitive measures to verify ovulation.

Due to the apparent lack of compliance with the methodology by this particular demographic of young women and the small number of participants, it is possible that the results are not entirely reflective of the BMSW device’s performance. Hence, piloting the methodology on a demographic more likely to follow the methodology correctly could be of value. For instance, studies could involve groups who are highly motivated to monitor ovulation, such as women attempting to conceive or who have presented with infertility. Moreover, when considering BMSW’s potential as a research tool in reproductive health and menstrual cycle research, the device’s galvanic skin receptor sensor could be applicable in menopause research related to vasomotor symptoms.

### Conclusions

This study demonstrated the importance of evaluating bio-measures collected using mobile monitoring devices by comparison with standard methods. It revealed a relatively poor correlation between BMSW and oral thermometer temperature readings and suggested that BMSW is unlikely to detect an upward shift in basal body temperature. Participant behavior suggested poor compliance in the use of BMSW for basal temperature measurement and that the basal body temperature method may not be suitable for use in unselected samples of young women. Our findings point to the need for simple, low-cost, self-administered methods for monitoring the menstrual cycle that are appealing to and accepted by young women.

## Abbreviations

BA: Bland-Altman
BBT: basal body temperature
BMSW: BodyMedia SenseWear
HPV: human papillomavirus
MTM: mean temperature method
OCP: oral contraceptive pill
VACCINE: Vaccine Against Cervical Cancer Impact and Effectiveness Study
YFHI: Young Female Health Initiative
